# Criminal Mania

**Published:** 1896-04-11

**Authors:** F. M. F. Skene


### Criminal Mania.

By F. M. F. Skene.

### I.—ELASTIC THEORIES.

The existence of criminal mania, as it is understood at the present day, may be considered one of the discoveries of the nineteenth century. In the days of our grandfathers, Justice, whether seated on the judge's bench or on the scarce less formidable throne of public opinion, followed hard and fast on the commission of crime with condign punishment, and troubled itself uncommonly little as to the motives which prompted the evil deeds or the mental condition of the individuals who perpetrated them. In fact, the departed judges and juries of these times seemed to think it decidedly preferable that a few innocent persons should be hanged than that a single criminal should escape. At all events, they appear to have acted habitually on the theory that crime could never under any circumstances be excusable. A reaction from this uncompromising severity was inevitable. A system of judicial discrimination was gradually forced on the administrators of the law, by the occurrence of cases presenting inexplicable features which could not be met by wholesale condemnation. It is now fully admitted in the courts of justice, as well as in general society, that there exists, apart from ordinary insanity, a criminal tendency which, however iniquitous in action, is yet compatible with innocence because maniacal. In these pages we have to deal with this matter mainly in its relation to the members of the medical profession, on whom it has entailed a most serious responsibility. But it is necessary for the elucidation of the subject, even in this respect only, that we should touch on some of its tendencies in a social point of view. The theory in question, taking vigorous root from its recognition in the law courts, has spread far and wide, till it has permeated all classes in this country with various results which seem to be not without certain subtle dangers to the wellbeing of the community. We have long since thoroughly accepted the terms dipsomania and kleptomania with all that they imply; and without having yet had recourse to the discreet veil of the delicate Greek idiom, there is no doubt that we have extended the protection of an irresponsible mania which is at once innocent and criminal to a variety of other delinquencies, such as murderous violence, brutal cruelty, systematic lying, and many more, including a long list of offences springing from unbridled passion, of which we will spare our readers the details. In fact, it may safely be said that the whole code of the moral law is embraced by the protecting shield of this elastic theory, and that there is no breach of any one of its enactments which may not be pronounced simply the innocent action of a person afflicted with the criminal malady. Probably many of our readers could recall some instance in their own recollection, where this convenient whitewash has been applied to the misdeeds of persons whose position in society renders their offences against recognised laws of morality a matter of surprise and dismay to their equals in birth and education. As a general rule, criminals of a lower class only get the benefit of a doubt as to their sanity when their cases are investigated in a court of justice, the world in general being quite content to assume that their undesirable actions are the result of their natural depravity. But it is not often that a malefactor of gentle birth is credited with the responsibility of his own nefarious acts, when they seem to be incompatible with his rank and station.

Now the tendency of this principle, especially when taken in connection with the doctrine of heredity, cannot be mistaken. It tends to the obliteration of the lines between right and wrong, to the perverting of that instinctive recognition of good as opposed to evil which in every variety of form is found to be inherent in the human mind. That this is the inevitable result of the theory on society in general cannot be questioned, and it is equally certain that it has the further effect on persons of good position who are disposed to criminal acts, of removing the restraint which a dread of disgrace to themselves or their friends might have imposed upon them. It may be argued by some that if the existence of criminal mania is an indisputable fact, these results cannot be avoided, although they may be deplored. This position we consider to be wholly untenable, and we are anxious to prove that it is so, especially in the interests of members of the medical profession, to whom it is a matter of vital importance. A somewhat careful study of the subject has convinced us that while a considerable amount of genuine evil-doing escapes scot free under the name of criminal mania, there are certain unmistakable symptoms of the true mental disease by which it may be distinguished from the real or excusable guilt that is sheltered under its disguise. We shall endeavour to point these out in our next article, illustrated by cases that have come under our notice, and we hope before concluding the series to deal with some of the more serious aspects of medical responsibility in this matter, as well as with certain dangers to classes of the community which are involved in it, and have been too long overlooked.

Before entering on these details, however, we must draw attention to the fact that there is one form of supposed criminal mania which is simply a transparent fiction that deceives no one—in the great majority of cases. Self-murder is a crime in the eye of the law; and the ancient penalties, still unrepealed, with which it was to be visited in the mode of the suicide's burial, as well as regard for the feelings of surviving friends, has engendered the undeviating rule that a verdict of temporary insanity is to be given by the coroner's jury on all such inquests. In rare cases the plea may be a true one, but in the epidemic of suicide which has set in of late years by far the greater proportion of persons so disposing of themselves have taken care to leave proofs, before committing the fatal act, that they were in perfect possession of their senses.

